# Regarding the Focus on Metrics in the Residency Application Process

**DOI:** 10.15694/mep.2018.0000141.1

**Published:** 2018-07-11

**Authors:** Richard Prayson

**Affiliations:** 1 Cleveland Clinic

**Keywords:** grades, tests, medical school metrics, applying for postgraduate training

## Abstract

This article was migrated. The article was marked as recommended.

No Abstract was necessary for this article.

## Perspective

More recently, some medical schools have experimented with moving away from grades, utilizing a pass/fail approach to segments of the curriculum, particularly in the first few years of school.  The benefits of this approach are myriad and are documented in the literature.  Rohe et al demonstrated that pass-fail grading in first year courses reduced student stress, improved student mood and facilitated group cohesion and collegiality ( Rohe et al. 2006).  There is a tendency for grades to engender extrinsic motivation in learners instead of more desired intrinsic motivation ( Forsyth, 2002).  Grades tend to decrease student interest in what they are learning, create preferences for easier tasks and reduce the quality of student thinking ( Kohn, 2011).  One study suggested that using a pass-fall approach to grading in year 2 of medical school, although associated with a slight decrease in performance on preclinical exams administered by the school, did not impact performance on the USMLE step 1 examination ( McDuff et al. 2014).  Spring and colleagues concluded in their literature review that pass-fail grading did not adversely impact objective academic performance and it enhanced student well-being ( Spring et al. 2011).  They also noted, however, that residency program directors believe that pass-fail grading is disadvantageous to students in the residency application process ( Spring et al, 2011).

During this last residency application cycle, I had a number of medical students approach me with concerns and stories about how they believed the lack of grades and a rank was adversely impacting how their applications were being considered by residency program directors.  These students are part of a small 5 year program focused on training students who have an interest in becoming physician scientists ( Dannefer and Henson, 2007).  During their medical school training, there are no tests administered, grades given, ranking generated or honor’s society awarded during any of the 5 years in the program.  The program utilizes a competency –based portfolio system as its principle means of assessment. Students regularly receive narrative feedback regarding their performance throughout and are asked to review how they are doing with respect to milestones that have been developed for each competency, based on their level of schooling.  Students are asked to reflect on their feedback, to identify targeted areas for improvement and to construct learning plans to address areas for improvement and to monitor their progress with subsequent feedback.  The goal is to foster reflective practice and to help students internalize this approach to self-improvement, which they will hopefully employ for the rest of their careers.

Since the beginning of the program, there has been ongoing anecdotal issues voiced by students regarding the impact of this assessment approach on the residency application process.  This seems to have increased in more recent years as programs are relying more on metrics to wade through increased numbers of applications. Students have indicated that some program directors have told them that they have no way of knowing how they did in school without grades/rank/honor’s designation; as a result, their application was simply set aside.  After all, there are plenty of applicants who have many or all of the other metrics. So, why struggle with the application that does not. Some residency programs use a scoring rubric that takes into account board scores, rank, grades, honor society status; without many of those parameters, missing metrics result in a low score using these rubrics.  Students have described situations in which they have called programs to followup on the status of their application to be told that they were not being invited because of the lack of grades or rank and that someone from the school needed to or should have called to explain the situation, even though it is delineated in the Deans letter (MSPE letter).  Even when interviewed, most students indicate that they commonly are asked lots of questions about the program’s assessment system, challenging the student to explain, “How do I know if you are clinically competent to take care of patients?”  The lack of other school generated metrics often results in undue emphasis being placed on the only metrics that do exist, the board exam scores.  This further increases the stress level in preparing for what is now viewed as a very high stakes test which are, at best, imperfect measures of a limited aspect of medical knowledge and reasoning and are not a reliable predictor of one’s ability to be a good physician.

So, how does one address these issues, given that the situation is not likely to change in the near future?  First, it is important to keep perspective.  The majority of residency programs are open to this assessment approach, particularly given the more recent move of residency assessment toward competencies and milestones; the approaches are not too dissimilar.  Most program directors, I believe, take a somewhat holistic look at the entire application.  Students need to be prepared to discuss the portfolio assessment approach, how it works and what they perceive as the benefits.  Each year, students report being asked a disproportionate number of questions about the program and it’s assessment process; often these questions take up a good portion of some interviews and most arise out of a true interest in understanding and learning about portfolios.  This is a good thing! Thirdly, students need to be a bit proactive in following up when rejected for an interview or when they have not heard anything form a program in a reasonable period of time; gentle inquiries have sometimes resulted in finding out that they were screened out because of missing metrics and this provides an opportunity to explain and educate.  Sometimes, this results in an invitation.  At the end of the day, our students, by doing well in residency and practicing the skills of self-reflection and self-improvement as residents, demonstrate that there are other ways to approach assessment.  In the words of Albert Schweitzer, “Example is not the main thing in influencing others.  It is the only thing.”

## Take Home Messages

There are many advantages to being in a medical school environment in which traditional grading metrics are not utilized. There are, however, implications for the student in applying to postgraduate or residency training where these metrics seem to be heavily relied upon to screen applications.

## Notes On Contributors

The contributor is Director of Student Affairs and the program’s career advisor. The program has no tests, grades or class rank ever generated; in lieu of these metrics, a portfolio assessment system is used.
