# Supplementary material for: Regarding the Focus on Metrics in the Residency Application Process
Source: MedEdPublish (2016). 2018 Jul 11;7:141. [Version 1] doi: 10.15694/mep.2018.0000141.1 (PMC10701825; doi:10.15694/mep.2018.0000141.1)
Supplement: Peer review history

# Reviewer report: Reviewer response for version 1

**DOI:** 10.21956/mep.19614.r29158

**Published:** 2018-07-12

**Authors:** P Ravi Shankar

**Affiliations:** 1 American International Medical University

This review has been migrated. The reviewer awarded 4 stars out of 5 This article raises a number of interesting questions. Traditionally we have relied on metrics and numbers for grading students and the assumption was the higher the scores the better the students. Over the years there has been a trend toward objectivity in assessment and away from judgment. We have tried to standardize the assessment process and break it down to manageable chunks. Assessment has moved away from the messy, often chaotic world of real life practice toward a standard simulated environment. This increases objectivity and reduces inter-rater or examiner variation but the downside may be that the assessment becomes more detached from real-life practice. Competency-based assessment and portfolios are becoming more common. This article highlights the challenges faced by students from a medical school which does not use numbers in the grading process. Faced with increasing number of applicants it is common for residency directors to choose an arbitrary cut off mark in a high stakes examination below which they will not consider applicants. This article will be of interest to a wide body of medical educators.

Reviewer Expertise:

NA

No decision status is available

# Reviewer report: Reviewer response for version 1

**DOI:** 10.21956/mep.19614.r29159

**Published:** 2018-07-11

**Authors:** Ronald M Harden

**Affiliations:** 1 AMEE

This review has been migrated. The reviewer awarded 4 stars out of 5 As demonstrated in the updated Ottawa consensus report on selection , to be publishes shortly in Medical Teacher, huge progress has been made on the selection of students for medical studies. Continuing effort is needed on the selection for training posts after students complete their undergraduate studies. Some of the problems are highlighted in this personal view which merits reading. Reference is made to portfolio assessment. When we introduced the portfolio as a key assessment instrument in the final qualifying examination in Dundee we had no difficulty in finding clinicians to serve as examiners as they identified reading a students portfolio and talking with the student about it was valuable in identifying the students they wished to appoint as junior doctors on their firm.

Reviewer Expertise:

NA

No decision status is available

# Reviewer report: Reviewer response for version 1

**DOI:** 10.21956/mep.19614.r29157

**Published:** 2018-07-11

**Authors:** Subha Ramani

**Affiliations:** 1 Harvard Medical School, Brigham and Women's Hospital

This review has been migrated. The reviewer awarded 4 stars out of 5 The authors make very important points regarding grades, or lack thereof, and potential effects on residency selection. If the pass-fail system promotes internal motivation and collaboration rather than competition among students, then should medical education not be hailing this approach? However, the confusion it may cause among program directors when selecting applicants for interviews is real. The top programs are typically intent on recruiting the top candidates and grades of “Honors” and “High Honors” make the selection process simpler when pursuing large volumes of applications. Having said that, I agree that it is important to be able to distinguish the more outstanding students from their peers in the pass / fail system and what domains they are outstanding in. Orientation of program directors and core educators on selection committees is critical so that they are able to effectively and efficiently review applications through a holistic lens. Scores and grades may not make the most motivated and reflective practitioners, but the system needs to be trained in this new approach.All medical educators involved in selection of trainees will find that this perspective stimulates reflection on the process.

Reviewer Expertise:

NA

No decision status is available
